# Field assessment of current and improved surveillance traps for fruit flies (Diptera: Tephritidae) in Australia

**DOI:** 10.1093/jee/toaf085

**Published:** 2025-04-22

**Authors:** Geoffrey W Brown, Melissa L Starkie, Elizabeth V Fowler, Mark J Blacket, Jane E Royer, David G Mayer, Natalia M Souza, Jodie Cheesman, Brendan Missenden, Mitchell Irvine, Mark K Schutze

**Affiliations:** Queensland Department of Primary Industries, Dutton Park, QLD, Australia; Queensland Department of Primary Industries, Dutton Park, QLD, Australia; Queensland Department of Primary Industries, Dutton Park, QLD, Australia; Agriculture Victoria Research, Bundoora, VIC, Australia; Queensland Department of Primary Industries, Dutton Park, QLD, Australia; Queensland Department of Primary Industries, Dutton Park, QLD, Australia; Queensland Department of Primary Industries, Dutton Park, QLD, Australia; Queensland Department of Primary Industries, Dutton Park, QLD, Australia; Queensland Department of Primary Industries, Dutton Park, QLD, Australia; Queensland Department of Primary Industries, Dutton Park, QLD, Australia; Queensland Department of Primary Industries, Dutton Park, QLD, Australia

**Keywords:** Dacini, Lynfield, Paton, real-time PCR, Steiner

## Abstract

Exotic fruit fly (Diptera: Tephritidae) surveillance in Australia predominantly relies on male-lure trapping. We assessed the performance of 3 traps currently used in Australian fruit fly surveillance: Lynfield, Modified Steiner, and Paton; against 3 improved versions: Enhanced Steiner, Enhanced Paton, and Enhanced Paton-10 mm. Laboratory trials revealed existing traps failed to exclude rain, and drained poorly, which guided our trap modifications. These modified traps were field-tested across 2 seasons and 4 locations in tropical and subtropical areas, with trap efficacy measured by total flies trapped, quality of fly DNA by real-time PCR, and weatherability observations. During the dry season, the Enhanced Paton trap outperformed all other traps in terms of fruit fly catch rates, a trend that continued in the wet season. While there was no discernible variation in DNA quality among flies caught by the 6 trap types, wet trap contents negatively affected DNA quality, with the incidence of wet trap catches influenced by trap design. No wet flies were observed in the Enhanced Paton trap, a result of the modifications made, which included a 3° entrance tube with a 42° angled roof. Overall, the Enhanced Paton trap proved to be a superior alternative to existing designs, offering higher fly capture rates and better-quality specimens for both morphological and molecular identification.

## Introduction

Dacine fruit flies (Diptera: Tephritidae: Dacinae) are economically damaging pests to fruit and vegetable industries worldwide (White and Elson-Harris 1992), with over a quarter of described fruit fly species (Doorenweerd et al. 2018) associated with commercial crops (Hancock et al. 2000). Damage to fresh produce is due to direct larval damage and secondary infection following oviposition (Bateman 1972, Clarke et al. 2011), hence early detection and eradication is critical in protecting fruit industries and trade.

**Fig. 1.**
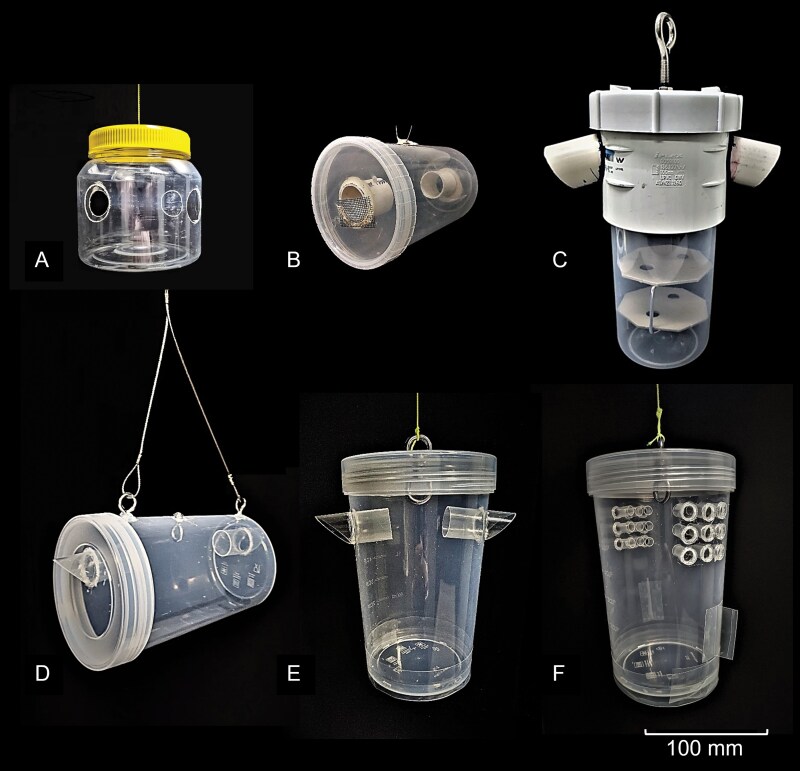
Six fruit fly trap designs that were compared in this study. Existing traps currently used for surveillance in Australia: A) Lynfield; B) Modified Steiner; C) Paton; and new design modifications developed in this study: D) Enhanced Steiner; E) Enhanced Paton; and F) Enhanced Paton-10 mm.

Early detection of exotic fruit flies in Australia relies heavily on monitoring via a surveillance trapping grid (Department of Agriculture Fisheries and Forestry 2023). Trapping is dacine-specific (Metcalf and Metcalf 1992) as males of more than 50% of species are strongly attracted to chemical compounds used to bait traps (Doorenweerd et al. 2018, Clarke 2019). Fruit fly surveillance in Australia is undertaken using dry bucket-style traps that are either horizontally or vertically oriented (Plant Health Australia 2024). In comparison to other trapping methods, dry traps are operationally simpler to set up, are multiuse, require less maintenance (Cowley et al. 1990, International Atomic Energy Agency 2003), and provide better quality insect specimens for identification, including for fruit flies (Plant Health Australia 2018). Within Australia, 3 dry trap designs are used to monitor for fruit fly incursions: Lynfield (Cowley et al. 1990) (Fig. 1A); Modified Steiner (Hooper and Drew 1978) (Fig. 1B); and Paton (Huxham 2002) (Fig. 1C). All 3 traps have differing justifications for their design and implementation: the Lynfield trap was developed as a cheaper and more efficient alternative to the sticky Jackson trap (Cowley et al. 1990); the Steiner trap (Steiner 1957) was developed as a cheaper alternative to bell-shaped invaginated glass traps; and McPhail traps (Newell 1936), with later modifications made to prevent sample loss (then referred to as the modified Steiner as described by Drew and Hooper (1981)); and the Paton trap was designed to preserve samples in areas that experience monsoonal rain and winds (Dominiak et al. 2016, Plant Health Australia 2018). Published trap comparisons have focused on comparing attractant and trap combinations (Hooper and Drew 1978), or sticky versus dry designs (O’Loughlin et al. 1983, Cowley et al. 1990) rather than direct comparisons of currently used traps (Meats et al. 2002, Dominiak et al. 2003, Dominiak and Nicol 2010). Given the variation in the design of traps in use, and the importance of trap efficacy, direct comparison of existing designs is essential.

Quick and efficient sample diagnosis is crucial for all early detection surveillance systems. Fruit fly trap catches are typically identified using morphological methods, which can be time-consuming due to the presence of nontarget by-catch (Doorenweerd et al. 2018) and poor sample quality due to exposure to environmental factors. Morphological identification of fruit flies is highly reliant on color patterns (Plant Health Australia 2018, Clarke 2019) and therefore, protection from environmental variables whilst in the trap is crucial (Fowler et al. 2024). Additionally, if a morphological result is inconclusive, specimen preservation is essential for confirmatory molecular diagnosis, especially for morphologically cryptic taxa (Charbonnel et al. 2023).

The objective of this study was to directly evaluate the 3 major trap designs currently used in Australian fruit fly surveillance programs: the Lynfield, Modified Steiner, and Paton traps. Specifically, as water is the primary driver of specimen degradation (morphological and molecular) (Nakahama et al. 2019, Martoni et al. 2021), we focused on the capacity of traps to exclude and drain water, and orientate out of the wind, and evaluated these factors under controlled laboratory conditions. Our findings were used to modify existing horizontal and vertical bucket-style trap designs for follow-up field trials. Our main aim was to develop a trap design that (i) catches at least as many flies as existing traps and (ii) maintains and preserves better quality specimens suitable for both morphological identification and DNA-based diagnostic approaches.

## Materials and Methods

### Traps

Traps tested in this study were designs currently used for fruit fly surveillance in Australia: Lynfield trap (Fig. 1A); Modified Steiner trap (Fig. 1B); Paton trap (Fig. 1C); and modified designs: Enhanced Steiner trap (Fig. 1D); Enhanced Paton trap (Fig. 1E); and Enhanced Paton-10 mm trap (Fig. 1F). As the Lynfield and Paton traps are both similar vertical bucket-style designs, we aimed to produce a single vertical style to replace both these designs, and similarly, a single horizontal design using our modifications.

**Fig. 2.**
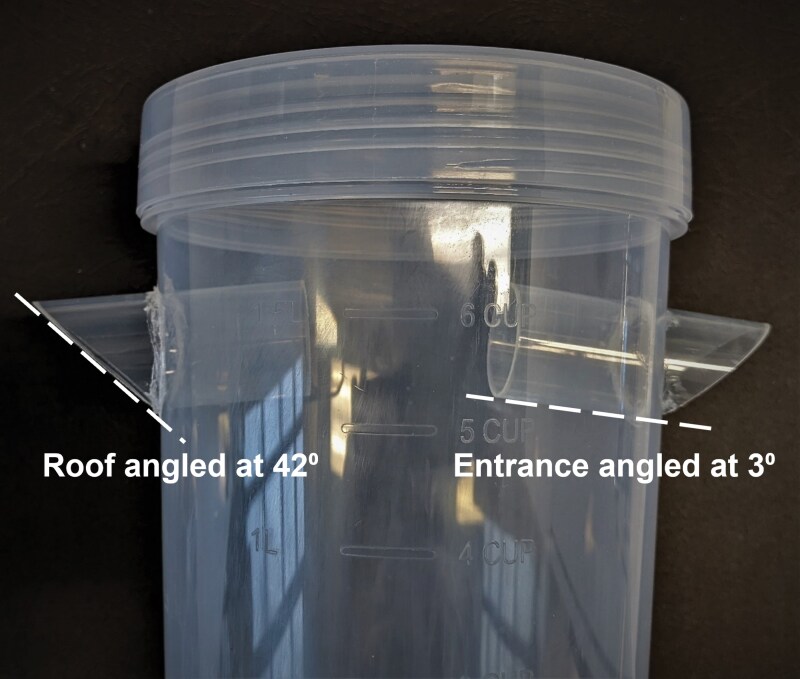
Design elements of the Enhanced Paton trap. Entrance tubes were designed to tilt 3° into the body of the trap to create a rain protecting cover with an optimal angle of 42°.

The new modified traps were designed around clear 2-liter round polypropylene containers (205 mm (H) × 133 mm (Ø), Anko, China). Entrances for the Enhanced Steiner and Enhanced Paton traps were made using clear polypropylene tubes, 27 mm internal diameter, 1 mm wall thickness, with the overall length of each being twice the internal diameter of tube (i.e. 54 mm). One end of each entrance tube was cut at 45° to form a “roof” over the section extending out of the trap. Each tube is tilted upwards slightly (2° to 3°) into the body of the trap to create a rain protecting cover with an angle of 42° to 43° (Fig. 2). The optimal angle for protection from water was calculated using the inverse tangent formula for right angled triangles: $\theta =tan^{-1}\left(dropletspeed/windspeed\right)$, and parameters for the smallest raindrop (0.5 mm diameter) (American Meteorological Society 2012) as this size has the greatest chance of being blown into a trap. The entrances for the Enhanced Paton-10 mm trap consist of 9, clear polypropylene tubes (10 mm internal diameter, 20 mm long) inserted on each side, with the holes flush with the outer surface of the trap.

**Fig. 3.**
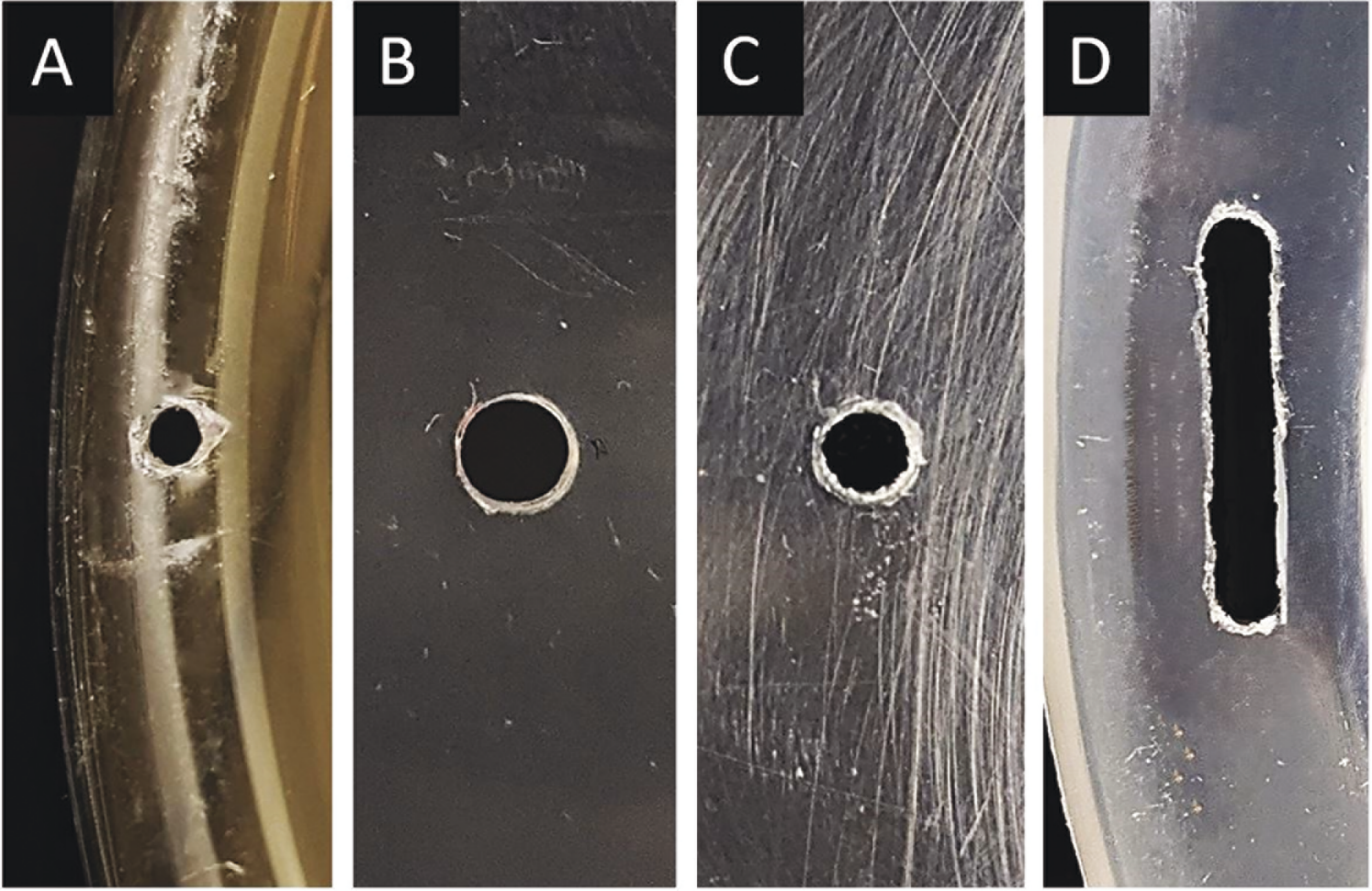
Comparison of drainage hole sizes in 5 different designs of fruit fly traps: A) 1.7 mm diameter hole in Lynfield trap; B) 3.0 mm diameter hole in Modified Steiner trap; C) 2.5 mm diameter hole in Paton trap; and D) 2.0 × 12.0 mm slot in the Enhanced Steiner and Enhanced Paton traps.

The Enhanced Steiner trap included 2 anchor points on the upper surface to create an incline when hanging to direct any water that enters the trap to flow down towards a 2 × 12 mm drainage slot (Fig. 3C) at one end. Adhesive foam strips (5 mm wide × 5 mm high) were attached under the container and around the drainage slot to direct water flowing over the trap away from the drainage hole.

The Enhanced Paton and Enhanced Paton-10 mm traps each have a single anchor point on top for hanging the traps, and 4 drainage slots in the base (again, each 2 mm wide × 12 mm long) (Fig. 3C). Clear adhesive tape (“Bear” All Weather Specialty Tape) was wrapped around the lower exterior of these traps to form a drip edge extending 10 mm past the bottom of the base to direct water away from the drain holes. A wind vane made from the clear adhesive tape (each 48 × 20 mm) was also attached to each side of the Enhanced Paton-10 mm trap.

### Trap Evaluation of Wind Movement and Drainage Performance

Trap movement in an artificial airstream and the water-draining capacity of 6 different traps were tested, with 5 samples of each trap evaluated in 5 separate trials. The speed at which traps rotated their entrance holes away from the wind ie at a 90° angle to the wind direction, was measured, along with recordings of trap behavior at different wind speeds. Based on a falling rain droplet speed of 2.06 m/s (Gunn and Kinzer 1949), the calculated horizontal wind speed required to blow rain into these entrance tubes is 2.21 m/s. Traps that rotated at wind speeds below 2.21 m/s, were considered good performers. Artificial wind was generated under controlled environmental conditions in a laboratory setting using a variable-speed axial-flow fan (Click 40 cm FT-40MD pedestal fan, Bunnings Australia) positioned at one end of a vented room. Traps were suspended in the airstream with their entrances facing into the wind. The airspeed at which the traps rotated, causing their entrances to turn away from the wind, was measured at the trap position using an Airflow LCA6000 anemometer (TSI Incorporated, USA).

To assess water drainage, a 10 ml syringe fitted with 1.0 mm ID tubing was used to add water to the traps. The tubing was inserted through the entrance hole, and water was added at the center of the trap base. Water was added slowly until drainage began. The volume of water added before draining started, and the amount remaining in each trap after drainage had ceased, were recorded.

### Trapping Logistics

**Fig. 4.**
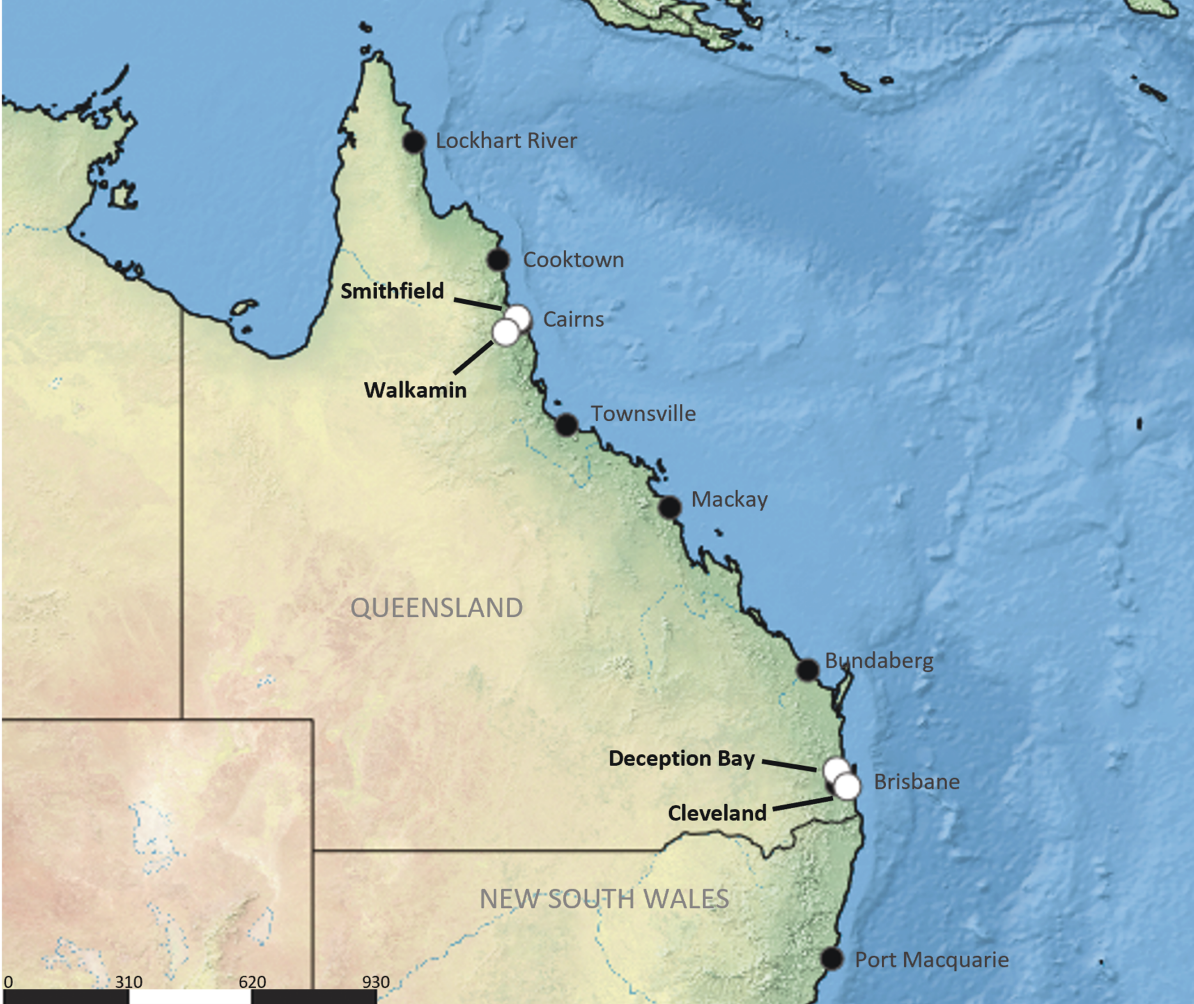
Map of Queensland, Australia, showing the 4 locations (indicated in white) where fruit fly trap designs were trialed over dry and wet seasons: Cleveland and Deception Bay located in the subtropical South East; Smithfield and Walkamin located in the tropical Far North. Map generated using SimpleMappr (https://www.simplemappr.net)

We tested the existing and new trap designs at 4 locations spanning 2 climatic zones (tropical and subtropical) in Queensland. The climatic zones were classified using a modified Köppen classification system for Australia (Australian Bureau of Meteorology 2023). Two locations were situated near Brisbane in subtropical South East Queensland: the QDPI Redlands Research Facility (Cleveland), and a private property in Deception Bay; and 2 locations near Cairns in tropical Far North Queensland: James Cook University (Smithfield) and the QDPI Research Facility in Walkamin (Fig. 4). The selection of Queensland locations was based on 3 key considerations: (i) this state presents some of the most challenging environmental conditions in Australia due to its subtropical and tropical climates (ie providing hot and humid conditions); (ii) much of Australia’s ongoing fruit fly surveillance is concentrated in this region; and (iii) Queensland has the greatest diversity and abundance of fruit flies among all Australian states or territories (Drew 1989, 2004) (ie higher populations provide a good baseline of trap efficacy).

Trapping occurred over 2 distinct periods: from 27 August to 22 October 2021 (dry season) and from 25 January to 5 April 2022 (wet season). The 2 trapping periods were selected to evaluate the performance of the trap designs under different seasonal conditions, specifically milder and drier versus hotter and wetter conditions (Supplementary Table S1).

All traps were baited with Q Fly Wick (Bugs for Bugs Pty Ltd, Australia) containing cue-lure (4-(*p*-methoxyphenyl)-2-butanone) (1 ml per wick) and contact insecticide maldison (malathion) (0.5 ml per wick). Traps were hung in a shady position from a tree branch (approx. 1.5 m above the ground) and spaced at least 50 m apart. Gloves were used for handling wicks to avoid contamination of other surfaces with the lure. Tree Guard nondrying glue (Horticulture Trade Corporation International Pty Ltd, Victoria, Australia) was applied to trap attachment points to reduce predator/scavenger access to traps.

Table 1 summarizes the trapping locations, periods, rotations, and trap designs used. Briefly, traps were deployed for a 2-wk rotation period which followed a Youden square design (an incomplete randomized Latin-square), where each trap was rotated to a new site after each clearance (ie 4 rotations for the dry season and 5 rotations for the wet season). The north Queensland locations consisted of 1 monoculture and 1 rainforest location, while the south Queensland locations consisted of 1 urban backyard and 1 mixed horticulture location. Following each rotation, flies were collected, lures replaced, and traps relocated. Predefined sites were chosen within each location, with one trap of each design set up at each location. The sites remained the same for the entirety of the dry and wet season trapping periods, except in Walkamin, where a different mango orchard was used for the wet season trial. An additional site was added to all locations for the wet season due to the inclusion of a sixth trap design (Enhanced Paton-10 mm).

**Table 1.** Summary of trapping periods, locations, number of sites within each location, together with number of 2-wk rotations used to test current and new fruit fly trap designs. Trap locations in Queensland included Cleveland (mixed horticulture) and Deception Bay (urban backyard) in the subtropical southeast, and Smithfield (rainforest) and Walkamin (monoculture) in the tropical far north of the state. Trapping occurred over 2 distinct periods: from 27 August to 22 October 2021 (dry season) and from 25 January to 5 April 2022 (wet season)

Trapping period	Trap location	Number of sites per location	Number of rotations	Trap designs tested (*n* = 1 at each location)
Dry season (8 wk)	Cleveland Deception Bay Walkamin Smithfield	5	4	Lynfield Modified Steiner Paton Enhanced Steiner Enhanced Paton
Wet season (10 wk)	Cleveland Deception Bay Walkamin Smithfield	6	5	Lynfield Modified Steiner Paton Enhanced Steiner Enhanced Paton Enhanced Paton-10 mm

Individual trap catches were manually counted and the total number of dacine flies were recorded alongside observations of sample condition (wet, dry, or scavenged). All dacine fruit flies were counted, but not differentiated by species or sex, as only a male attracting lure was used (Clarke 2019). By-catch was easily distinguishable, and not included in the counts.

### DNA Extraction and Real-time PCR to Evaluate DNA Quality

Crude DNA was isolated from entire trap catches using the nondestructive method outlined in Fowler et al. (2023). Briefly, flies from an entire catch were gently submerged in HotSOAK buffer (1.0 ml per approx. 50 flies; 12.5 mM NaOH; 5 mM Tris–HCl; 0.5 mM EDTA, pH 8.0) using a spatula and incubated at 75 °C for 10 min. Following this, 1.0 ml of the crude fly DNA preparation (ie lysate) was transferred to a fresh 1.5 ml tube and stored at −20 °C for further analysis. To evaluate DNA quantity and quality, the samples were tested without further processing using published Dacini-specific cytochrome c oxidase I (COI) primers (LCO1490-mod, Dac-COI-r; Krosch et al. 2020) in a real-time high-resolution melt PCR assay using a Rotor-Gene Q Real-time PCR cycler (QIAGEN). All samples were tested in triplicate, as batched runs, but blinded for location and trap type, and in random order. A synthetic double stranded DNA (dsDNA) positive control (gBlock) was designed herein to estimate DNA quantity in trap samples, and no-template controls were included in every run (for real-time PCR methods, primer and gBlock details, see Supplementary Material S2 and Table S3).

Real-time PCR data was generated using Rotor-Gene Q Series Software (Qiagen, Version 2.3.5, Build 1) with the baseline fluorescence threshold set to 0.02 RFU (relative fluorescent units) for amplification. For melt curve analysis the threshold was set at 0.25 dF/dT. Samples with melt curve peaks that did not match the expected peak profile relative to the positive control sample (ie incorrect melt temperature or unusual peak shape) were excluded as nonspecific and the PCR repeated. Replicate data were averaged and exported to Excel (Microsoft Office, Version 2308) for collation and sorting according to sample identification.

### Data Analysis and Statistical Methods

Generalized linear models (GLMs) (McCullagh and Nelder 1989) were used for data analyses, using Genstat v21.1 (2022). The adopted GLMs used a Poisson distribution with a log link for trap counts, binomial distribution with a logit link for binary wet trap data, and normal distribution with an identify link for wind speed, water retention and Ct data.

All analyses initially fitted the experimental design factors (Location, Season, Site, and Rotation), followed by trap design and its interactions. Nonsignificant interactions (*P* > 0.05) were omitted from the final models. Fisher’s protected least significant difference testing was applied to the adjusted means.

## Results

### Movement of Traps in Wind

Observations from the wind speed rotation experiment found that not all trap designs ceased rotating in the wind, due to differences in manufacturing. As mentioned earlier, our benchmark for rotation was set at a maximum speed of 2.21 m/s; higher than this, traps that did not rotate would allow rain to enter the trap. The wind speed at which each trap design rotated their entrance holes out of the direction of oncoming wind is summarized in Table 2. The Modified Steiner and Enhanced Steiner traps started rotating at lower wind speeds (1.33 ± 0.15 and 1.18 ± 0.21 m/s, respectively), while the Paton and Enhanced Paton designs required higher speeds (3.07 ± 0.44 and 2.07 ± 0.15 m/s, respectively). The Steiner and Paton traps both rotated such that their entrance holes faced away from the wind. The Lynfield trap, with its 4 large (30 mm diameter) entrance holes always had at least 1 hole or partial hole facing the wind when it rotated.

**Table 2.** Mean windspeed (m/s) at which traps rotated their entrance holes at 90° from simulated wind direction (standard error: 0.26 for Lynfield; 0.12 for the other traps)

Trap design	Lynfield	Paton	Modified Steiner old	Modified Steiner new	Enhanced Paton	Enhanced Steiner
Mean	3.94^a^	3.07^b^	1.57^d^	1.33^de^	2.07^c^	1.18^e^
No. of replicates	1*	4*	5	5	5	5

N.B.: *of the 5 technical replicates tested, not all replicates ceased rotating.

Means with a common superscript are not significantly different (*P *= 0.05).

Of the 5 Lynfield traps sampled, we observed irregular spacing of the entrance holes around the sides of the trap, as well as differences (as much as 11 mm) in height of the entrance holes in relation to the base of the trap, and inconsistent size of the drainage holes. Other observations included poorly positioned attachment points and irregular alignment of the drainage holes in Modified Steiner traps, and inconsistent lengths of Paton trap entrance tubes; factors which affected the results during drainage and wind trials.

### Water Retention

The new Enhanced traps, featuring 2 × 12 mm drainage slits, performed better than all other traps with round drainage holes (Table 3 and Fig. 3) across both the drainage ability, and water retention lab trials. The amount of water added to the trap before drainage began was significantly different across the 5 designs tested (*F* = 36.0; df = 6, 29; *P *< 0.001). Similarly, the water retained in the trap after drainage was complete, was also significant among the designs tested (*F* = 8.47; df = 6, 29; *P* < 0.001).

**Table 3.** Summary of 5 trap design drainage characteristics; mean water holding capacity before drainage initiated; and mean volume of water retained after drainage had completed.

Trap design (*n* = 5)	Number of drain holes	Drain hole size (mm)	Water holding capacity (ml) (*n *= 5)	Water retention (ml) (*n *= 5)
Lynfield	3	Ø 0.9–1.8	17.8^b^	6.8^c^
Modified Steiner	2	Ø 3.0	9.4^a^	3.7^b^
Paton	4	Ø 2.5	39.0^c^	7.0^c^
Enhanced Steiner	1	Slot, 2.0 × 12.0	5.8^a^	0.7^a^
Enhanced Paton	4	Slot, 2.0 × 12.0	3.7^a^	1.0^ab^
Pooled standard error			2.05 min rep.; 1.87 max rep.	1.00 min rep.; 0.92 max rep.

N.B.: the Enhanced Paton-10 mm had the same drainage hole design as the Enhanced Paton, so was not tested here.

Means with a common superscript are not significantly different (*P *= 0.05).

### Field Trial

#### Numbers of Flies Trapped

**Fig. 5.**
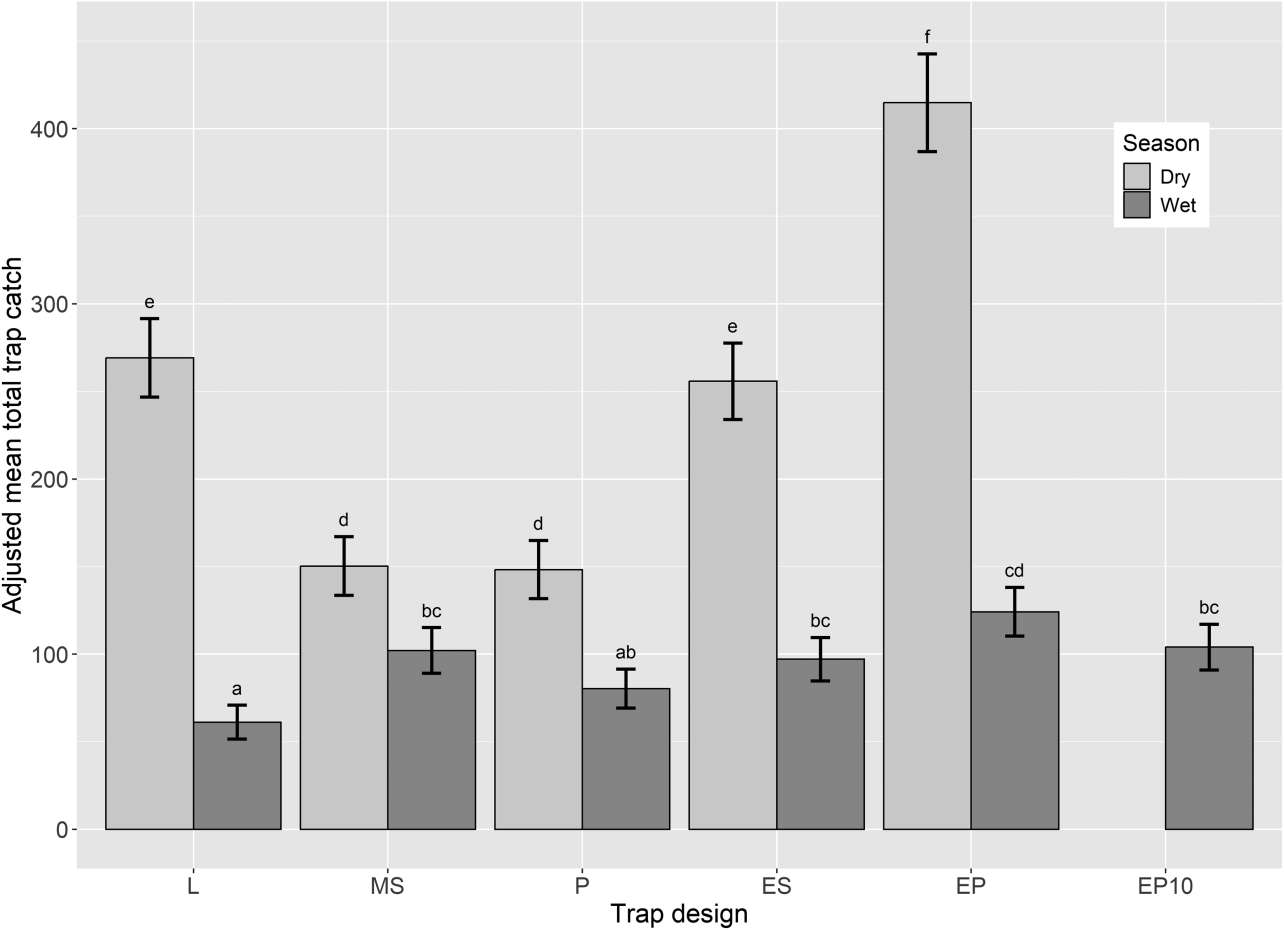
Mean adjusted numbers of fruit flies (± standard error) caught by each trap design across all locations for dry and wet seasons in Queensland. Existing traps currently used for surveillance in Australia: L = Lynfield; MS = Modified Steiner; and P = Paton. New trap designs developed in this study: ES = Enhanced Steiner; EP = Enhanced Paton; EP10 = Enhanced Paton-10 mm (developed following results of the dry season trial and deployed only in the wet season). Means with a common superscript are not significantly different (*P* = 0.05).

The number of flies trapped differed significantly across the 4 locations of Cleveland, Deception Bay, Smithfield, and Walkamin (*F* = 137.1; df = 3, 106; *P* < 0.001); as well as between the dry and wet seasons (*F* = 218.7; df = 1, 106; *P *< 0.001) (Supplementary Tables S4 and S5). There was a significant difference in the adjusted mean number of flies trapped among the trap designs tested (*F* = 7.06; df = 5, 106; *P *< 0.001) (Fig. 5 and Supplementary Table S6), and importantly, season was the only factor that significantly interacted with trap design (*F* = 4.60; df = 4, 106; *P* < 0.01), confirming that the traps performed comparably across locations, sites, and rotations. Across both seasons and in all locations except one, the Enhanced Paton trap consistently outperformed all other traps, including the 3 currently used in surveillance programs—Lynfield, Modified Steiner, and Paton traps. The only exception was during the wet season in Walkamin, where the Enhanced Steiner trap captured 191 more flies than the Enhanced Paton trap (*n* = 515 and 324, respectively). During the dry season, the Enhanced Paton trap caught more flies than all other traps, while in the wet season, it also outperformed the other traps, with notably higher captures than the Lynfield and Paton traps. When evaluating surveillance traps currently in use, the Lynfield trap caught more flies than both the Modified Steiner and Paton traps during the dry season (Fig. 5). Conversely, in the wet season, the Lynfield trap exhibited the lowest efficacy, catching fewer flies than all other traps.

#### Specimen Integrity

Although instances of scavenged flies and wet trap contents were relatively low across treatments (21 and 11 of 200 total clearances, respectively), the occurrence of wet trap catches was significantly influenced by both season (*F* = 4.72; df = 1, 189; *P* < 0.05) and, more importantly, trap design (*F* = 2.97; df = 5, 189; *P* < 0.05). The interaction (Season * Trap) was not significant. Not surprisingly, a higher number of samples were observed as wet during the wet season (*n* = 9) compared to the dry season (*n* = 2). In the dry season trial, the Lynfield trap was the only design with wet flies, with occurrences observed in 2 rotations. During the wet season, the Lynfield trap presented wet flies in 5 rotations. In contrast, both the Enhanced Paton and the Enhanced Paton-10 mm outperformed all other designs, effectively excluding or draining water throughout the trial. The Enhanced Paton and Enhanced Paton-10 mm traps kept flies dry at Deception Bay despite major flooding rain during the wet season trial.

Scavenging by ants was the predominant cause of fly removal, even with the utilization of the Tree Guard barrier. However, indications of vertebrate predation (ie feces) was noted inside traps at Smithfield, once during the dry season trial for the Enhanced Steiner design and on 2 occasions in the wet season—once for the Lynfield trap and once for the Enhanced Paton design.

#### DNA Quality

Season, Trap and Season * Trap did not significantly impact cycle threshold (Ct) values. Comparing locations, Smithfield displayed significantly lower quality DNA compared to other locations (*F* = 9.80; df = 3, 158; *P* < 0.001). DNA quality was significantly different for dry and wet samples (*F* = 14.59; df = 1, 156; *P* < 0.001), with adjusted mean Ct value of 22.5 (standard error = 0.22) for dry flies, compared to 26.2 (standard error = 1.16) for wet flies (Table 4). Comparing DNA quality in flies caught by different trap designs across both dry and wet seasons showed no significant interaction (*F* = 0.42; df = 4, 158; *P* > 0.5). For further details on amplification and DNA quality, see Supplementary Tables S7 to S9.

**Table 4.** Comparison of DNA quality from trap catch contents that were observed to be dry or wet. A high cycle threshold (Ct) value indicates a lower concentration of fly DNA in the sample. Ct values > 40 indicates samples that failed to amplify and produce a readable value; these values were not used in the means analysis. Trap catches from field trials conducted in subtropical and tropical areas of Queensland. Different letters indicate significant (*P* < 0.001) difference between adjusted mean Ct values for wet and dry trap samples)

Condition of flies	Number of traps	Number of Ct values > 40	Adjusted mean Ct values	Standard error	Ct value range
Dry	184	9	22.5^a^	0.217	17.3–34.8
Wet	11	4	26.2^b^	1.155	22.7–35.2

## Discussion

We directly compared 3 trap designs currently used in fruit fly surveillance in Australia, alongside 3 proposed new designs. By identifying limitations in the existing designs, we developed and tested modifications under multiseason, multiclimatic scenarios in Queensland, Australia. The result is the Enhanced Paton trap, a well-rounded design that incorporates the best features of the existing traps: the weatherability of the Paton, the high catch rates of the Modified Steiner, and the cost-effective assembly of the Lynfield.

Our dry season results show that the Enhanced Paton trap caught the most flies, followed by the Lynfield and Enhanced Steiner traps. However, in the wet season, while the Enhanced Paton trap was still the best performer, the Lynfield trap caught fewer flies than all other traps. Disparities in the Lynfield trap catches between dry and wet seasons may be attributed to the large entrance holes. While these large entrance holes allow easy access for flies, they also increase the likelihood of flies escaping before encountering the insecticide, a problem encountered in other trapping systems (Lasa et al. 2014). Additionally, Lynfield traps are known to perform poorly in windy conditions, with documented instances of samples being blown out of traps (Kean et al. 2023). The large entrance holes likely contributed to the increased instances of wet flies, as our laboratory trials showed that the Lynfield design cannot rotate all openings away from the wind to prevent rain intrusion. Overall, our findings highlighted the need for a more weatherproof trap design.

Large entrance holes allowed scavenging insects to feed on trap contents during our field trials. The entry of scavenging insects is unfortunate but often unavoidable. While predator entry can be the result of poor trap maintenance (ie plant foliage in contact with the trap or insufficient Tree Guard) (Plant Health Australia 2018), it is not an indicator of poor trap design. Despite every precaution, flying insects can still enter traps and feed on the contents (Katsoyannos 1994). Our observations of predation during the first field trial led us to develop an Enhanced Paton trap with narrower, 10 mm internal diameter, entrance tubes (the Enhanced Paton-10 mm). Use of narrower entrance tubes for fruit fly traps was previously described by Tan (1985) and has been successfully used in the design of the Lucitrap for sheep blowflies (*Lucilia cuprina*) (Green et al. 1994, Urech et al. 2009). Although the Enhanced Paton-10 mm trap was not evaluated during the dry season, there was no significant difference in mean trap catch numbers when compared to the Enhanced Paton design during the wet season. Our findings suggest comparable performance to the Enhanced Paton however, this needs further evaluation.

When examining DNA quality of trap contents, we recorded high Ct values in several trap catches indicating poor sample quality. This degradation could be attributed to factors such as exposure to water, environmental impacts such as high temperatures and humidity, solar radiation, predation, and microbial activity; many of which have been linked to DNA degradation (Lindahl 1993, Mandrioli 2008, Zimmerman et al. 2008). Previous studies on mixed-species samples have shown inconsistent relationships between the number of target flies and Ct values (Jarrett et al. 2010), with DNA degradation often caused by environmental moisture (Jarrett et al. 2010), a finding also reflected here. Maintaining dry trap catches and preserving DNA integrity is crucial, especially for downstream applications like PCR, sequencing, and high-throughput genomic approaches (Ballare et al. 2019, Martoni et al. 2021, Fowler et al. 2024). The Lynfield trap presented with the majority of wet trap samples, while both Enhanced Paton designs had no instances of wet trap contents. Our findings support the use of the more weatherproof Enhanced Paton trap design to better preserve sample integrity.

Our study evaluated the performance of the Enhanced Paton trap across 4 locations and multiple sites, with 2 wk replicates at each site. Although our data spans only a limited timeframe, the Enhanced Paton consistently outperformed or matched the performance of all other traps across all variables. This suggests that factors like seasonality may be less relevant to its effectiveness. The success of biosecurity surveillance programs depends heavily on the quality of traps used to detect exotic incursions, and the Enhanced Paton trap offers significant advantages in this regard. It not only captures more flies than currently used traps but is also cost-effective, simple to construct, and easy to deploy in the field, with its low cost due to a straightforward design and inexpensive, readily available materials. Additionally, its design protects trap contents from degradation by excluding rain, ensuring accurate downstream morphological and molecular diagnostics. Future research could explore scaling up production to minimize the need for labor-intensive manual assembly. Leveraging technologies like 3D printing could further enhance production efficiency, consistency, and optimization of the trap for wider applications. Given the growing threat of exotic insect incursions, the increasing demand for more robust surveillance systems, and the need for preserving samples for high-throughput molecular genomic analyses, the value of the Enhanced Paton trap is clear. Improved trapping technologies not only benefit biosecurity, but also have the potential to improve surveillance in broader scientific applications, such as biodiversity research and ecological monitoring (Novotny et al. 2005).

## Supplementary material

Supplementary material is available at *Journal of Economic Entomology* online.

toaf085_suppl_Supplementary_Tables_S1-S9_Materials_S2
