# Supplementary material for: Field assessment of current and improved surveillance traps for fruit flies (Diptera: Tephritidae) in Australia
Source: J Econ Entomol. 2025 Apr 22;118(3):1344–53. doi: 10.1093/jee/toaf085 (PMC12167848; doi:10.1093/jee/toaf085)
Supplement: toaf085_suppl_Supplementary_Tables_S1-S9_Materials_S2 [file toaf085_suppl_supplementary_tables_s1-s9_materials_s2.docx]

Field assessment of current and improved surveillance traps for fruit flies (Diptera: Tephritidae) in Australia

GW Brown, ML Starkie, EV Fowler, MJ Blacket, JE Royer, DG Mayer, NM Souza, J Cheesman, B Missenden, M Irvine, MK Schutze (2024)

Supplementary material

**Supplementary Table S1.** Typical climatic variables experienced across the four trapping locations in Queensland, Australia. Cleveland and Deception Bay are located near Brisbane in the subtropical south east; Smithfield and Walkamin are located near Cairns in the tropical far north of the state. Data taken from Australian Bureau of Meteorology (2023) sites nearest to the trapping locations.

|  | Cleveland | Deception Bay | Smithfield | Walkamin |
| --- | --- | --- | --- | --- |
| August-October (dry season) |  |  |  |  |
| Mean monthly number of wet days | 3.0 | 5.6 | 5.1 | 2.5 |
| Mean (decile 5) monthly rainfall | 44.4 mm | 44.9 mm | 21.6 mm | 7.4 mm |
| Average monthly temperature range | 11.2–23.4 °C | 13.9–23.6 °C | 19.0–28.2 °C | 15.0–27.1 °C |
| January-April (wet season) |  |  |  |  |
| Mean monthly number of wet days | 4.9 | 10.0 | 15.6 | 12.2 |
| Mean (decile 5) monthly rainfall | 117.9 mm | 93.8 mm | 321.9 mm | 145.4 mm |
| Average monthly temperature range | 18.6–27.7 °C | 20.2–27.7 °C | 23.1–30.7 **°**C | 19.7–28.5 **°**C |

**Supplementary Material S2.** Methods for conducting insect-specific cytochrome c oxidase I (COI) real-time PCR assay.

PCR reactions (20 µL) contained 4.0 µL 5x Solis Biodyne HOT FIREPol® EvaGreen® HRM Mix (no ROX) (Integrated Sciences, Australia), 0.25 µM of each forward and reverse primers (Supplementary Table 1), 2.0 µL of DNA sample, and 13.0 µL nuclease-free water. Cycling was completed on a Rotor-Gene RGQ Real-time PCR cycler (Qiagen, Australia) using the following conditions: 12 min at 95 °C; 40 cycles of 95 °C for 15 s, 53 °C for 20 s, 72 °C for 20 s (acquiring to green channel), followed by a final melt step ramping from 70–99 °C rising by 1 °C each step (acquiring to HRM channel).

A synthetic double stranded DNA (dsDNA) positive control (gBlock; Integrated DNA Technologies, Iowa, USA) matching 355bp of the 5’ end of the *Bactrocera neohumeralis* COI gene (GenBank accession #: MZ520733.1) (Supp. Table S3) was used to estimate DNA quantity in test samples (Fowler et al. 2024). The gBlock was prepared at 1 x 10^10^ copies/µL and serial 10-fold dilutions made to 10 copies/µL. The limit of detection of this assay was determined to be <20 copies of COI per reaction. Standards were included in every run at three concentrations ranging from 2 x 10^4^ to 2 x 10^6^ copies/reaction allowing the software to automatically calculate DNA copy number in test samples.

**Supplementary Table S3.** Primer and Gene Block sequences used in the Insect-COI real-time PCR. Primer binding sites in gBlock are underlined; inserted “ccc” bases are in lowercase. Primer references also included.

| Primer name | Nucleotide sequence | Reference |
| --- | --- | --- |
| LCO1490-modF | TYTCAACAAATCATAAAGATATTGG | (Krosch et al. 2020) |
| Dac-COI-r | GTTCAACCTGTACCVGCYCCGTTTTC | (Krosch et al. 2020) |
| Dacini_COI_gBlock | cccTTTCAACAAATCATAAAGATATTGGcccAACTTTATATTTTATCTTCGGAGCCTcccGAGCAGGAATAGTCGGGACATCCCTTcccAGAATTTTAGTCCGGGCTGAACTAcccGGACACCCCGGGGCATTAATCGGAGcccACGATCAGATTTATAATGTAATTGTcccAACAGCCCATGCTTTCGTAATGATTcccTTCTTTATAGTTATACCAATTATAAcccTTGGTGGTTTCGGAAACTGGCTTcccGTTCCTTTAATATTAGGTGCGCCCGAcccTATAGCATTCCCACGAATAAATAATcccATAAGATTTTGATTACTACCCCCTTCCCTTACACTACTcccATTAGTGAGAAGTATAGTAcccGAAAACGGAGCTGGTACAGGTTGAACccc | (Fowler et al. 2024) |

**Supplementary Table S4. D**ry season trial (August-October): Total number of trapped fruit flies across four locations and five rotations for five trap designs. Trap locations in Queensland encompassed Cleveland and Deception Bay in the subtropical south east, and Smithfield and Walkamin in the tropical far north. Traps were deployed for a two-week rotation period. Following each rotation, flies were collected, lures replaced, and traps relocated to a new site within the same location.

| **Trap location** | **Trap design** | **Total number of flies trapped in each rotation** | | | | **Total number of flies / trap design** |
| --- | --- | --- | --- | --- | --- | --- |
|  |  | **1** | **2** | **3** | **4** |  |
| **Cleveland** | Lynfield | 68 | 86 | 57 | 68 | **279** |
|  | Modified Steiner | 47 | 67 | 118 | 140 | **372** |
|  | Paton | 47 | 26 | 101 | 112 | **286** |
|  | Enhanced Steiner | 50 | 52 | 140 | 276 | **518** |
|  | Enhanced Paton | 16 | 54 | 154 | 310 | **534** |
| **Deception Bay** | Lynfield | 71 | 170 | 261 | 224 | **726** |
|  | Modified Steiner | 33 | 115 | 258 | 140 | **546** |
|  | Paton | 134 | 215 | 121 | 82 | **552** |
|  | Enhanced Steiner | 83 | 137 | 710 | 414 | **1,344** |
|  | Enhanced Paton | 80 | 227 | 257 | 1,122 | **1,686** |
| **Smithfield** | Lynfield | 1,104 | 1,116 | 635 | 181 | **3,036** |
|  | Modified Steiner | 69 | 431 | 62 | 405 | **967** |
|  | Paton | 399 | 367 | 322 | 163 | **1,251** |
|  | Enhanced Steiner | 689 | 527 | 460 | 107 | **1,783** |
|  | Enhanced Paton | 1,456 | 471 | 1,500 | 341 | **3,768** |
| **Walkamin** | Lynfield | 21 | 9 | 151 | 73 | **254** |
|  | Modified Steiner | 103 | 48 | 119 | 235 | **505** |
|  | Paton | 45 | 60 | 68 | 96 | **269** |
|  | Enhanced Steiner | 24 | 43 | 248 | 121 | **436** |
|  | Enhanced Paton | 42 | 85 | 319 | 173 | **619** |
|  | **Total** |  |  |  |  | **19,731** |

**Supplementary Table S5.** Wet season trial (January-April): Total number of trapped fruit flies across four locations and five rotations for six trap designs. Trap locations in Queensland encompassed Cleveland and Deception Bay in the subtropical south east, and Smithfield and Walkamin in the tropical far north. Traps were deployed for a two-week rotation period. Following each rotation, flies were collected, lures replaced, and traps relocated to a new site within the same location.

| **Trap location** | **Trap design** | **Total number of flies trapped in each rotation** | | | | | **Total number of flies / trap design** |
| --- | --- | --- | --- | --- | --- | --- | --- |
|  |  | **1** | **2** | **3** | **4** | **5** |  |
| **Cleveland** | Lynfield | 98 | 72 | 42 | 64 | 99 | **375** |
|  | Modified Steiner | 32 | 29 | 25 | 83 | 81 | **250** |
|  | Paton | 59 | 80 | 36 | 42 | 62 | **279** |
|  | Enhanced Steiner | 76 | 51 | 59 | 55 | 90 | **331** |
|  | Enhanced Paton | 85 | 122 | 126 | 38 | 106 | **477** |
|  | Enhanced Paton-10 mm | 72 | M | 49 | M | 186 | **307** |
| **Deception Bay** | Lynfield | 45 | 26 | 41 | 78 | 1 | **191** |
|  | Modified Steiner | 62 | 29 | 68 | 79 | 9 | **247** |
|  | Paton | 45 | 52 | 28 | 57 | 28 | **210** |
|  | Enhanced Steiner | 47 | 38 | 42 | 82 | M | **209** |
|  | Enhanced Paton | 66 | 18 | 51 | 67 | 54 | **256** |
|  | Enhanced Paton-10 mm | 39 | 18 | 53 | 35 | 23 | **168** |
| **Smithfield** | Lynfield | 177 | 56 | 7 | 83 | 149 | **472** |
|  | Modified Steiner | 288 | 406 | 131 | 93 | 229 | **1,147** |
|  | Paton | 176 | 218 | 135 | M | 222 | **751** |
|  | Enhanced Steiner | 205 | 265 | 110 | 34 | 244 | **858** |
|  | Enhanced Paton | 80 | 580 | 111 | 204 | 558 | **1,533** |
|  | Enhanced Paton-10 mm | 51 | 301 | 367 | 217 | 215 | **1,151** |
| **Walkamin** | Lynfield | 33 | 46 | 36 | 22 | 57 | **194** |
|  | Modified Steiner | M | 87 | 109 | 38 | 11 | **245** |
|  | Paton | 33 | 38 | 110 | 121 | 94 | **396** |
|  | Enhanced Steiner | 91 | 102 | 105 | 118 | 99 | **515** |
|  | Enhanced Paton | 99 | 86 | 62 | 52 | 25 | **324** |
|  | Enhanced Paton-10 mm | 63 | 112 | 78 | 87 | 20 | **360** |
|  | **Total** |  |  |  |  |  | **11,246** |

M = Missing value as traps had either fallen or contents entirely predated upon.

**Supplementary Table S6.** Comparison of adjusted mean numbers of fruit flies captured by each trap design in tropical and subtropical Queensland, per rotation, across both the dry (August-October) and wet (January-April) seasons for the six trap designs. Under Fisher’s protected least significant difference testing, means sharing a common letter are not significantly different (at *P* = 0.05).

| **Trap design** | **Dry season**  **Mean trap count** | **Standard error** | **Wet season**  **Mean trap count** | **Standard error** |
| --- | --- | --- | --- | --- |
| Lynfield | 269.2^e^ | 22.4 | 61.2^a^ | 9.6 |
| Modified Steiner | 150.3^d^ | 16.8 | 102.1^bc^ | 13.0 |
| Paton | 148.3^d^ | 16.7 | 80.3^ab^ | 11.1 |
| Enhanced Steiner | 255.8^e^ | 21.9 | 97.1^bc^ | 12.6 |
| Enhanced Paton | 414.8^f^ | 27.9 | 124.2^cd^ | 13.9 |
| Enhanced Paton-10 mm* | * | * | 104.0^bc^ | 13.1 |

*Enhanced Paton-10 mm was deployed in the wet season trial only.

**Supplementary Table S7. D**ry season trial (August-October): Average insect COI Ct value (± standard deviation) obtained from real-time PCR analysis of DNA lysates of fruit flies trapped in each of the five trap designs. A high cycle threshold (Ct) value indicates a lower concentration of fly DNA in the sample. Trap locations in Queensland encompassed Cleveland and Deception Bay in the subtropical south east, and Smithfield and Walkamin in the tropical far north. Traps were deployed for a two-week rotation period. Following each rotation, flies were collected, lures replaced, and traps relocated to a new site within the same location.

| **Trap location** | **Average Ct value /**  **Trap location** | **Trap design** | **Ct values** | | | | **Average Ct value /**  **Trap design** |
| --- | --- | --- | --- | --- | --- | --- | --- |
|  |  |  | **Rotation 1** | **Rotation 2** | **Rotation 3** | **Rotation 4** |  |
| Cleveland | 22.1 ± 4.6 | Lynfield | 20.8 | 20.6 | 18.5 | 35.2 | 23.8 ± 7.7 |
|  |  | Modified Steiner | 19.7 | 22.3 | 17.9 | 24.2 | 21.0 ± 2.8 |
|  |  | Paton | 20.8 | 19.6 | 19.1 | 26.4 | 21.5 ± 3.3 |
|  |  | Enhanced Steiner | 19.7 | 19.9 | 23.2 | 25.5 | 22.1 ± 2.8 |
|  |  | Enhanced Paton | 17.3 | 19.7 | 19.0 | 32.0 | 22.0 ± 6.8 |
| Deception Bay | 22.7 ± 3.3 | Lynfield | 21.7 | 20.1 | 23.6 | No amp. | 21.8 ± 1.8 |
|  |  | Modified Steiner | 20.2 | 26.9 | 19.9 | 29.3 | 24.1 ± 4.8 |
|  |  | Paton | 21.3 | 27.7 | 19.6 | 21.5 | 22.5 ± 3.6 |
|  |  | Enhanced Steiner | 21.6 | 20.3 | No amp. | 28.5 | 23.5 ± 4.4 |
|  |  | Enhanced Paton | 22.1 | 20.1 | 19.5 | 25.1 | 21.7 ± 2.5 |
| Smithfield | 23.4 ± 4.3 | Lynfield | 25.6 | 20.8 | 22.7 | 22.1 | 22.8 ± 2.0 |
|  |  | Modified Steiner | 19.2 | 21.0 | 19.1 | No amp. | 19.8 ± 1.1 |
|  |  | Paton | 33.0 | 19.8 | 20.8 | 21.9 | 23.9 ± 6.1 |
|  |  | Enhanced Steiner | 31.5 | 19.6 | No amp. | 23.1 | 24.7 ± 6.1 |
|  |  | Enhanced Paton | 25.4 | 19.7 | 28.6 | 28.2 | 25.5 ± 4.1 |
| Walkamin | 22.0 ± 2.4 | Lynfield | 20.1 | 22.8 | 21.4 | 25.4 | 22.4 ± 2.3 |
|  |  | Modified Steiner | 25.8 | 19.7 | 22.9 | 25.0 | 23.4 ± 2.8 |
|  |  | Paton | 20.8 | 20.2 | 21.1 | 22.0 | 21.0 ± 0.7 |
|  |  | Enhanced Steiner | 18.8 | 19.5 | 23.8 | 23.3 | 21.4 ± 2.5 |
|  |  | Enhanced Paton | 20.3 | 19.7 | 20.3 | 27.1 | 21.8 ± 3.5 |

Ct value = number of cycles in real-time PCR assay required before amplification reaches the required threshold.

No amp. = No amplification of DNA indicating samples failed to produce a readable value (i.e., Ct value >40); these values were not used in the calculations of the means.

**Supplementary Table S8.** Wet season trial (January-April): Average insect COI Ct value (± standard deviation) obtained from real-time PCR analysis of DNA lysates of fruit flies trapped in each of the six trap designs. A high cycle threshold (Ct) value indicates a lower concentration of fly DNA in the sample. Trap locations in Queensland encompassed Cleveland and Deception Bay in the subtropical south east, and Smithfield and Walkamin in the tropical far north. Traps were deployed for a two-week rotation period. Following each rotation, flies were collected, lures replaced, and traps relocated to a new site within the same location.

| **Trap location** | **Average Ct value /**  **Trap location** | **Trap design** | **Ct values** | | | | | **Average Ct value /**  **Trap design** |
| --- | --- | --- | --- | --- | --- | --- | --- | --- |
|  |  |  | **Rotation 1** | **Rotation 2** | **Rotation 3** | **Rotation 4** | **Rotation 5** |  |
| Cleveland | 22.2 ± 1.5 | Lynfield | 23.3 | 25.5 | 25.6 | 21.3 | 23.4 | 23.8 ± 1.8 |
|  |  | Modified Steiner | 21.7 | 20.0 | 22.9 | 20.6 | 23.5 | 21.7 ± 1.5 |
|  |  | Paton | 23.8 | 21.2 | 21.0 | 20.3 | 21.9 | 21.6 ± 1.3 |
|  |  | Enhanced Steiner | 22.7 | 19.6 | 20.7 | 23.7 | 22.7 | 21.9 ± 1.7 |
|  |  | Enhanced Paton | 22.4 | 22.2 | 22.4 | 20.7 | 21.4 | 21.8 ± 0.7 |
|  |  | Enhanced Paton-10 mm | 22.9 | M | 22.0 | M | 21.2 | 22.0 ± 0.9 |
| Deception Bay | 21.8 ± 2.9 | Lynfield | 24.4 | 19.8 | 24.8 | 21.4 | 24.4 | 23.0 ± 2.2 |
|  |  | Modified Steiner | 23.8 | 20.0 | 25.5 | 20.5 | 19.7 | 21.9 ± 2.6 |
|  |  | Paton | 20.6 | 20.5 | No amp. | 21.3 | 19.4 | 20.5 ± 0.8 |
|  |  | Enhanced Steiner | 21.8 | 20.5 | No amp. | No amp. | M | 21.1 ± 0.9 |
|  |  | Enhanced Paton | 21.9 | 19.7 | 23.6 | 32.3 | 20.4 | 23.6 ± 5.1 |
|  |  | Enhanced Paton-10 mm | 21.2 | 19.9 | 18.3 | 21.4 | 18.9 | 19.9 ± 1.4 |
| Smithfield | 25.9 ± 4.1 | Lynfield | No amp. | 21.8 | 20.3 | 27.0 | 32.5 | 25.4 ± 5.5 |
|  |  | Modified Steiner | No amp. | 27.2 | No amp. | 30.3 | No amp. | 28.7 ± 2.2 |
|  |  | Paton | 21.1 | 21.3 | 21.0 | M | 24.7 | 22.0 ± 1.8 |
|  |  | Enhanced Steiner | 25.8 | 23.6 | 24.2 | 30.0 | 25.4 | 25.8 ± 2.5 |
|  |  | Enhanced Paton | 24.5 | 34.8 | No amp. | 30.6 | 26.6 | 29.1 ± 4.6 |
|  |  | Enhanced Paton-10 mm | 25.1 | 24.1 | No amp. | 31.4 | 21.4 | 25.5 ± 4.2 |
| Walkamin | 21.7 ± 1.5 | Lynfield | 24.4 | 23.6 | 22.7 | 21.3 | 20.5 | 22.5 ± 1.6 |
|  |  | Modified Steiner | M | 21.0 | 21.4 | 20.5 | 20.5 | 20.9 ± 0.4 |
|  |  | Paton | 22.5 | 22.3 | 19.6 | 22.0 | 21.0 | 21.5 ± 1.2 |
|  |  | Enhanced Steiner | 23.3 | 25.3 | 22.7 | 23.1 | 21.4 | 23.2 ± 1.4 |
|  |  | Enhanced Paton | 22.1 | 20.8 | 21.2 | 22.2 | 19.1 | 21.1 ± 1.2 |
|  |  | Enhanced Paton-10 mm | 18.7 | 22.0 | 21.5 | 23.2 | 20.2 | 21.1 ± 1.7 |

M = Missing value as traps had either fallen or contents entirely predated upon.

Ct value = number of cycles in real-time PCR assay required before amplification reaches the required threshold.

No amp. = No amplification of DNA indicating samples failed to produce a readable value (i.e., Ct value >40); these values were not used in the calculations of the means.

**Supplementary Table S9.** Comparison of adjusted mean Ct values from flies captured by six trap designs across the dry and wet seasons. A high cycle threshold (Ct) value indicates a lower concentration of fly DNA in the sample. Trap locations in Queensland encompassed Cleveland and Deception Bay in the subtropical south east, and Smithfield and Walkamin in the tropical far north. Adjusted mean proportions and their standard errors were estimated and protected least significant difference testing was conducted. The interaction of trap type by season was not significant (F = 0.42; DF = 4, 158; *P* = 0.79) (Fisher’s protected least significant difference test).

| **Trap design** | **Dry season**  **Mean Ct values** | **Standard error** | **Wet season Mean Ct values** | **Standard error** |
| --- | --- | --- | --- | --- |
| Lynfield | 22.7 | 0.745 | 23.5 | 0.664 |
| Modified Steiner | 22.2 | 0.745 | 22.5 | 0.723 |
| Paton | 22.2 | 0.721 | 21.4 | 0.683 |
| Enhanced Steiner | 22.6 | 0.771 | 23.2 | 0.701 |
| Enhanced Paton | 22.7 | 0.721 | 23.6 | 0.664 |
| Enhanced Paton-10 mm* | * | * | 21.9 | 0.702 |

*Enhanced Paton-10 mm was deployed in the wet season trial only.

Ct value = number of cycles in real-time PCR assay required before amplification reaches the required threshold. Samples with Ct values >40 failed to amplify DNA and were not used in the analysis.

**References**

**Australian Bureau of Meteorology**. *Climate statistics for Australian sites*. [Accessed June 21 2023]. **2023**. <http://www.bom.gov.au/climate/averages/tables/ca_qld_names.shtml>.

**Fowler EV, Starkie ML, Blacket MJ, Mayer DG, Schutze MK**. Effect of temperature and humidity on insect DNA integrity evaluated by real-time PCR. J. Econ. Entomol. **2024**.

**Krosch MN, Strutt F, Blacket MJ, Batovska J, Starkie M, Clarke AR, Cameron SL, Schutze MK**. Development of internal COI primers to improve and extend barcoding of fruit flies (Diptera: Tephritidae: Dacini). Insect Sci. **2020**:27:143–158.
